# Downregulation of Key Early Events in the Mobilization of Antigen-bearing Dendritic Cells by Leukocyte Immunoglobulin-like Receptor B4 in a Mouse Model of Allergic Pulmonary Inflammation

**DOI:** 10.1371/journal.pone.0057007

**Published:** 2013-02-19

**Authors:** Laura B. Fanning, Carolyn C. Buckley, Wei Xing, Rebecca G. Breslow, Howard R. Katz

**Affiliations:** From the Department of Medicine, Harvard Medical School, Boston, Massachusetts, United States of America, and the Division of Rheumatology, Immunology and Allergy, Brigham and Women's Hospital, Boston, Massachusetts, United States of America; Harvard Medical School, United States of America

## Abstract

Leukocyte Immunoglobulin-like Receptor B4 (LILRB4) null mice have an exacerbated T helper cell type 2 (Th2) immune response and pulmonary inflammation compared with *Lilrb4^+/+^* animals when sensitized intranasally with ovalbumin (OVA) and low-dose lipopolysaccharide (LPS) followed by challenge with OVA. Moreover, OVA-challenged *Lilrb4* ^−/−^ mice exhibit greater migration of antigen (Ag)-bearing dendritic cells (DCs) to lymph nodes and accumulation of interleukin 4- and interleukin 5-producing lymph node lymphocytes. The main objective of this study was to determine how the absence of LILRB4 leads to a greater number of DCs in the lymph nodes of Ag-challenged mice and increased lung Th2 inflammation. Mice were sensitized intranasally with PBS alone or containing OVA and LPS; additional cohorts were subsequently challenged with OVA. Expression of chemokine (C-C motif) ligand 21 (CCL21) in the lung was assessed immunohistologically. OVA ingestion and expression of LILRB4 and chemokine (C-C motif) receptor 7 (CCR7) were quantified by flow cytometry. Inhalation of OVA and LPS induced upregulation of LILRB4 selectively on lung Ag-bearing DCs. After sensitization and challenge, the lung lymphatic vessels of *Lilrb4* ^−/−^ mice expressed more CCL21, a chemokine that directs the migration of DCs from peripheral tissue to draining lymph nodes, compared with *Lilrb4^+/+^* mice. In addition, lung DCs of challenged *Lilrb4* ^−/−^ mice expressed more CCR7, the CCL21 receptor. The lungs of challenged *Lilrb4* ^−/−^ mice also contained significantly greater numbers of CD4+ cells expressing interleukin-4 or interleukin-5, consistent with the greater number of Ag-bearing DCs and Th2 cells in lymph nodes and the attendant exacerbated Th2 lung pathology. Our data establish a new mechanism by which LILRB4 can downregulate the development of pathologic allergic inflammation: reduced upregulation of key molecules needed for DC migration leading to decreases in Th2 cells in lymph nodes and their target tissue.

## Introduction

Dendritic cells (DCs) are among the earliest participants in the initiation of both protective and pathologic adaptive immunity and inflammation. In response to danger signals such as lipopolysaccharide (LPS), immature tissue DCs undergo an innate immune maturation process so as to degrade endocytosed antigen (Ag) and present peptides in association with highly expressed cell surface major histocompatibility molecules in concert with upregulated costimulatory molecules. This leads to expansion of Ag-specific effector T cells in tissue-draining lymph nodes (LNs), a hallmark of adaptive immunity [1]. An essential step in this process is the migration of tissue DCs to draining LNs, where Ag-bearing mature DCs attract and activate cognate Ag-specific T cells, leading to their proliferation, polarization, and migration from the LNs to the blood [2]–[4]. This process occurs not only in the initiation of immune responses, but also at the beginning of inflammatory responses that can occur upon re-exposure to Ag. For example, challenge of Ag-sensitized animals by Ag inhalation results in increased numbers of airway DCs and migration of the cells to the lung-draining LNs, particularly in the first 24 hours after challenge [5]–[8]. Similarly, there is a rapid increase in the number of DCs in the bronchial mucosa of individuals with allergic asthma in response to inhaled allergen challenge [9]. Lung DCs that respond to Ag challenge in sensitized animals play a significant role in increasing the number of pulmonary T cells and their ability to generate T helper cell type 2 (Th2) cytokines in response to Ag, as well as in the development of eosinophilic airway inflammation, goblet cell hyperplasia, and airway hyperresponsiveness [10]–[12]. Whereas much is known about the innate immune signals that initiate this process through the migration of Ag-bearing DCs to LNs, less is understood about control mechanisms that inhibit DC migration and the subsequent development of pathologic adaptive immune inflammation.

We have shown that the cell surface receptor Leukocyte Immunoglobulin-like Receptor B4 (LILRB4) is a potent inhibitory regulator of LPS-induced, neutrophil-dependent inflammation [13]–[15]. More recently, we discovered a regulatory role for LILRB4 on DCs [16] in a mouse model of Th2 pulmonary inflammation induced by inhalation sensitization with OVA and a low dose of LPS followed by inhalation challenge with ovalbumin (OVA) two weeks later [17]. Low doses of inhaled LPS also exacerbate pathologic Th2 airway responses in humans and augment influx of myeloid DCs into the airways of patients with allergic asthma [18], [19]. In our model, *Lilrb4* ^−/−^ mice have exacerbated Th2 pathology consisting of greater amounts of eosinophilia and epithelial goblet cell metaplasia in the lung, OVA-specific serum IgE, and production of Th2 cytokines by OVA-restimulated lung-draining LN cells [16]. We also found that expression of LILRB4 is selectively upregulated on OVA**^+^** DCs in the LNs during sensitization of *Lilrb4^+/+^* mice. Moreover, the LNs of OVA-challenged *Lilrb4* ^−/−^ mice have a significantly greater number of OVA**^+^** mature DCs that contain a greater amount of Ag, as well as more interleukin (IL) 4- and IL-5-producing lymphocytes, compared with *Lilrb4^+/+^* mice [16]. Upregulation of Ag uptake by airway DCs is associated with the progression of allergic airway inflammation [20]. The greater Th2 pathology of *Lilrb4* ^−/−^ mice is reiterated in *Lilrb4^+/+^* mice sensitized by inhalation of *Lilrb4* ^−/−^ DCs previously pulsed with OVA/LPS *in vitro* and challenged two weeks later by inhalation of OVA, compared with mice sensitized with *Lilrb4^+/+^* DCs, which indicates that LILRB4 expressed on DCs is sufficient to downregulate the detrimental allergic airway responses [16]. The mechanism by which LILRB4 decreases the number of Ag-bearing lung DCs that appear in the draining LNs after Ag challenge represents a fundamental control step in the development of allergic pulmonary inflammation. Hence, we sought to determine how LILRB4 regulates this aspect of DC pathobiology.

Chemokine (C-C motif) ligand 21 (CCL21) is a chemoattractant for DCs *in vitro* and plays a key role in regulating the migration of tissue DCs to draining LNs [21]–[27]. Upregulation of CCL21 on lymphatic endothelium can be a rate-limiting step in DC migration from peripheral tissue to draining LNs [28]. In the lung, CCL21 is located in perivascular lymphatic vessels [23]. A large body of evidence indicates that expression of chemokine (C-C motif) receptor 7 (CCR7; the receptor for CCL21) on DCs is essential for their entry into lymphatic vessels [22], [24], [27], [29]–[31], including lung DCs carrying inhaled OVA [32]. Upregulation of CCR7 expression on DCs accompanies maturation induced by LPS, tumor necrosis factor α (TNF-α), and other proinflammatory mediators [33]–[36]. We therefore hypothesized that the greater number of OVA**^+^** DCs in the LNs of OVA-challenged *Lilrb4* ^−/−^ mice was the result of enhanced migration of the cells from the lung to LNs. We now report that LILRB4 is selectively upregulated on OVA**^+^** DCs in the lungs of OVA/LPS-sensitized *Lilrb4* ***^+/+^*** mice when challenged with OVA, indicating that this effect, previously observed in the draining LNs [16], occurs first in the lung. We also show that expression of CCL21 on lung lymphatic vessels and CCR7 on OVA**^+^** lung DCs is increased after challenge of OVA/LPS-sensitized *Lilrb4* ***^+/+^*** and *Lilrb4* ^−/−^ mice, but the increases are significantly greater in the *Lilrb4* ^−/−^ strain. In accordance with the greater number of OVA**^+^** DCs and Th2 cells in the LNs of *Lilrb4* ^−/−^ mice [16], the number of Th2 cells in the lungs of Ag-challenged *Lilrb4* ^−/−^ mice is significantly greater than that of *Lilrb4* ***^+/+^*** animals. Our data reveal that LILRB4 downregulates the expression of two key molecules that induce the migration of Ag-bearing lung DCs to LNs, thereby attenuating Th2 cell accumulation in LNs and lung as well as ensuing pathologic inflammation.

## Methods

### Animals

*Lilrb4^+/+^* and *Lilrb4* ^−/−^ mice were generated on the BALB/c background as previously described [14]. Mice were maintained in a specific pathogen-free barrier facility. Female mice, 6–12 weeks old, were used for experiments. Mice were anesthetized for all intranasal instillations to minimize discomfort. The use of mice for these studies was reviewed and approved by the Animal Care and Use Committee of the Dana-Farber Cancer Institute (Protocol #03-139), and complied with the U.S. Public Health Service Policy on Humane Care and Use of Laboratory Animals, 2002.

### Sensitization and challenge protocol

Mice were lightly anesthetized with isoflurane and then sensitized intranasally with 50 µl of PBS alone or containing 100 µg of OVA (Grade V, Sigma-Aldrich, St. Louis, MO) and 100 ng of LPS (from E. coli 055:B5; Sigma-Aldrich #L4524) on days 0, 1, and 2 [17]. On day 14, mice were lightly anesthetized with isoflurane and challenged intranasally with 25 µg of OVA or Alexa Fluor 647 (AF)-OVA (Invitrogen) in 30 µl of PBS. As determined with the limulus amebocyte assay (Cambrex), a total dose of <1.5 ng of contaminating LPS per mouse was delivered by the OVA solutions during sensitizations and challenges. Mice were euthanized before analyses at the times indicated in the Results section.

### Lung mononuclear cell isolation

Lungs were perfused with 10 ml of ice-cold calcium- and magnesium-free Hanks' Balanced Salt Solution through the right ventricle, removed intact, and physically dissociated with a gentleMACS Dissociator (Miltenyi Biotec). Lung fragments were incubated with 500 U/ml collagenase (CLS-IV, Worthington Biochemical) and 0.02 mg/ml DNAse I (Sigma-Aldrich) at 37°C for 20 minutes with agitation at 200 rpm. Residual tissue fragments were allowed to settle for 5 min, and the cell suspension was removed and washed with 1 mM EDTA on ice. The residual tissue fragments were digested again with fresh collagenase/DNAse and processed the same way as the first digest. Cells were pelleted by centrifugation, resuspended in RPMI medium, and the two digests were pooled. Mononuclear cells were isolated from the interface of a Nycoprep 1.077 (Axis-Shield) gradient (300×g for 20 minutes at 4°C). Cells were washed with PBS, counted, and analyzed by flow cytometry.

### Flow cytometry

Cells were resuspended in PBS containing 0.5% fetal bovine serum (FBS) or bovine serum albumin (BSA) and 0.05% sodium azide at 4°C and incubated with anti-FcγRII/III monoclonal antibody (mAb) (BD Biosciences) and mouse IgG (Sigma) for 20 minutes on ice to block FcγR. Cells were then incubated with saturating concentrations of fluorochrome conjugated anti-CD4, CD11c (Biolegend), or LILRB4 [13] for 30 minutes at 4°C and washed by centrifugation as previously described [16]. For detection of CCR7, cells were incubated with mouse CCL19-human Fc (eBioscience) for 30 minutes at 4°C. Cells were washed by centrifugation, incubated with fluorochrome conjugated anti-human IgG for 20 minutes at 4°C, and washed again. For intracellular cytokine staining, cells were resuspended in media containing 50 ng/ml phorbol myristate acetate (Sigma) and 1 µM ionomycin (Calbiochem) and incubated for 2 hours at 37°C. Monensin (2.5 µM; Sigma) was then added, and the cells were incubated for 4 hours at 37°C, after which DNase I (50 µg/ml; Sigma-Aldrich) was added, and the cells were incubated for 15 minutes at 37°C. Cells were then resuspended in fixation buffer (eBioscience), incubated for 20 minutes in the dark at room temperature, washed twice by centrifugation with PBS containing 0.5% FBS or BSA and 0.05% sodium azide at 4°C, and washed once in permeabilization buffer (eBioscience) by centrifugation. Cells were resuspended in permeabilization buffer and incubated with anti-FcγRII/III mAb (BD Biosciences) and mouse IgG (Sigma-Aldrich) as described above. Cells were then incubated with saturating concentrations of fluorochrome conjugated anti-CD4 and anti-IL-4 or anti-IL-5 (Biolegend) in permeabilization/blocking buffer for 15 minutes in the dark on ice, and washed by centrifugation with permeabilization buffer. Cells were then incubated on ice in fresh permeabilization buffer for 10 minutes, centrifuged, and resuspended in PBS containing 0.5% FBS or BSA and 0.05% sodium azide. Data were acquired on a Becton Dickinson FACSCanto flow cytometer using FACSDiva acquisition software, and data were analyzed with FlowJo software (Treestar). Fluorescence compensation was set for each color such that there was no cross-talk between detection channels.

### Fluorescence immunohistology

Mice were euthanized by i.p. injection of pentobarbital and exsanguinated by cardiac puncture. Lungs were perfused with 10 ml of ice-cold calcium- and magnesium-free Hanks' Balanced Salt Solution through the right ventricle. A 22-gauge plastic cannula was inserted into the upper third of the trachea, and 0.4 ml of 4% paraformaldehyde was infused into the lungs. Lungs were removed intact, at the hila, placed in 4% paraformaldehyde, stored at 4°C, and embedded in paraffin blocks. Tissue sections were deparaffinized, rehydrated, and underwent Ag retrieval by incubation in Target Retrieval Solution (Dako; Cat. S1699) at 97°C for 30 minutes. Sections were then incubated in a mixture of goat anti-mouse CCL21 (R&D Systems) and rabbit anti-mouse lymphatic vessel endothelial receptor 1 (LYVE-1) (Cell Sciences) at 37°C for 1 h. The sections were washed and incubated in a mixture of Alexa Fluor 488-labeled donkey anti-goat IgG (1∶1000; Invitrogen), Alexa Fluor 594-labeled chicken anti-rabbit IgG (1∶1000; Invitrogen), and nuclear dye (Hoechst 33342, Invitrogen) at 37°C for 1 h. Sections were washed thoroughly with PBS and mounted with Fluoro-Gel (Electron Microscopy Sciences). Lung sections were viewed with an Eclipse 80i microscope (Nikon Instruments, Inc.), and photomicrographs were acquired with the Hamamatsu C10800 digital camera and HC Image software 1.1.3.1 (Hamamatsu Corp.).

#### Statistical analyses

Statistical significance was determined using Student's unpaired, two-tailed t-test or the Mann-Whitney test. P values<0.05 were considered to be significant.

## Results

### Expression of LILRB4 on lung DCs

**Figure 1:**
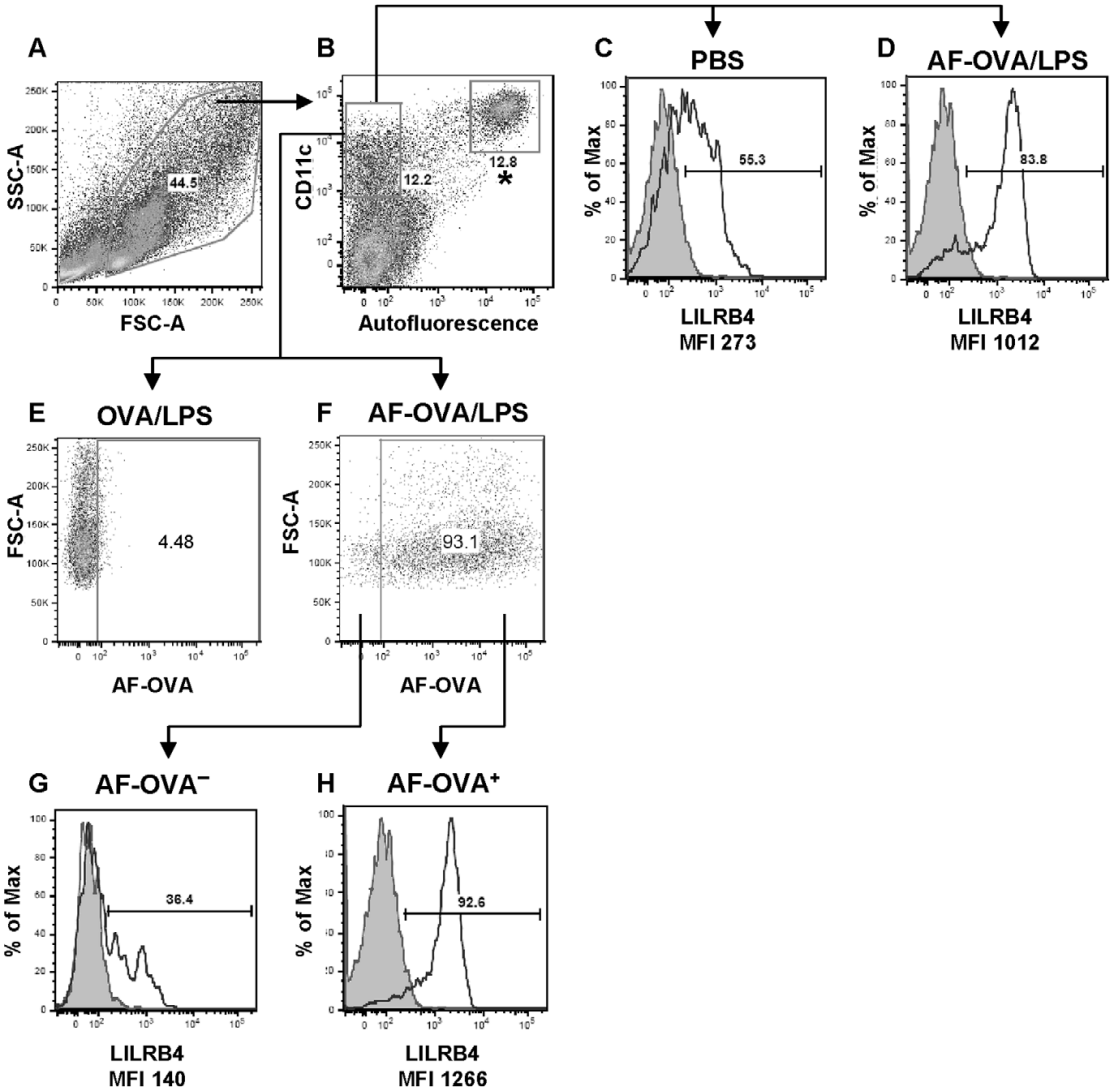
Expression of LILRB4 on lung DCs of *Lilrb4^+/+^* mice. Animals received PBS alone or containing 100 µg OVA and 100 ng LPS or 100 µg AF-OVA and 100 ng LPS intranasally. After 15 h, mice were euthanized, lungs were enzymatically digested, and mononuclear cells were obtained by density gradient centrifugation. Residual erythrocytes, dead cells, and debris were excluded in flow cytometric analysis by light scatter properties (A), and CD11c**^+^**/autofluorescence**^−^** cells (B) were analyzed for LILRB4 expression (C and D; shaded and open histograms are isotype control and anti-LILRB4 staining, respectively). Fluorescence compensation was set for each color such that there was no cross-talk between detection channels. Cells from mice treated with AF-OVA and LPS were further gated into AF-OVA**^−^** and AF-OVA**^+^** populations, as defined by cells from mice that received unlabeled OVA/LPS (E and F), and expression of LILRB4 was determined for each population (G and H). CD11c**^+^**/autofluorescence^+^ cells (* in B) were 100% AF-OVA**^+^**, and LILRB4 expression was significantly greater in mice that received AF-OVA compared with PBS (compiled data are reported in the Results section).

We previously reported that expression of LILRB4 is upregulated on LN DCs 18 hours after a single inhalation of 100 ug of OVA and 100 ng of LPS compared with inhalation of PBS in *Lilrb4^+/+^* mice [16]. The increase occurred selectively in OVA**^+^** DCs rather than in OVA**^−^** DCs. To determine whether that difference reflects the situation in the lung, *Lilrb4^+/+^* mice were given PBS alone or containing 100 µg OVA and 100 ng LPS or AF-labeled OVA and 100 ng LPS intranasally. After 15 h, mice were euthanized, their lungs were removed, and total cells were dispersed from the tissue by mechanical and enzymatic treatments. Mononuclear cells were then isolated by density gradient centrifugation. Any residual erythrocytes, dead cells, and debris in the mononuclear cell population were excluded in flow cytometric analysis by light scatter properties (Fig. 1A), and CD11c**^+^**/autofluorescence**^−^** cells (Fig. 1B) were analyzed for LILRB4 expression. In mice treated with PBS, LILRB4 was expressed on 55% of DCs with a mean fluorescence intensity (MFI) of 273, whereas in mice given OVA and LPS, 84% of DCs were LILRB4**^+^** with an MFI of 1012 (Figs. 1C and 1D). Cells from mice treated with AF-OVA and LPS were further gated into AF-OVA**^−^** and AF-OVA**^+^** populations, as defined by comparison with cells from mice that received unlabeled OVA/LPS (Figs. 1E and 1F), and expression of LILRB4 was determined for each population from mice that received AF-OVA (Figs. 1G and 1H). Whereas 38±0.6% of AF-OVA**^−^** DCs were LILRB4**^+^** with an MFI of 144±2.1 ± a significantly greater 91±1.2% of AF-OVA**^+^** DCs were LILRB4**^+^** (P<0.0001, *n* = 3) with a significantly greater MFI of 1267±53 (P<0.0001, *n* = 3). Thus, uptake of Ag by lung DCs in response to OVA and LPS is associated with increased expression of LILRB4, which is maintained on DCs in the lung-draining LNs [16].

### Effects of LILRB4 deficiency on CCL21 expression in the lung

**Figure 2:**
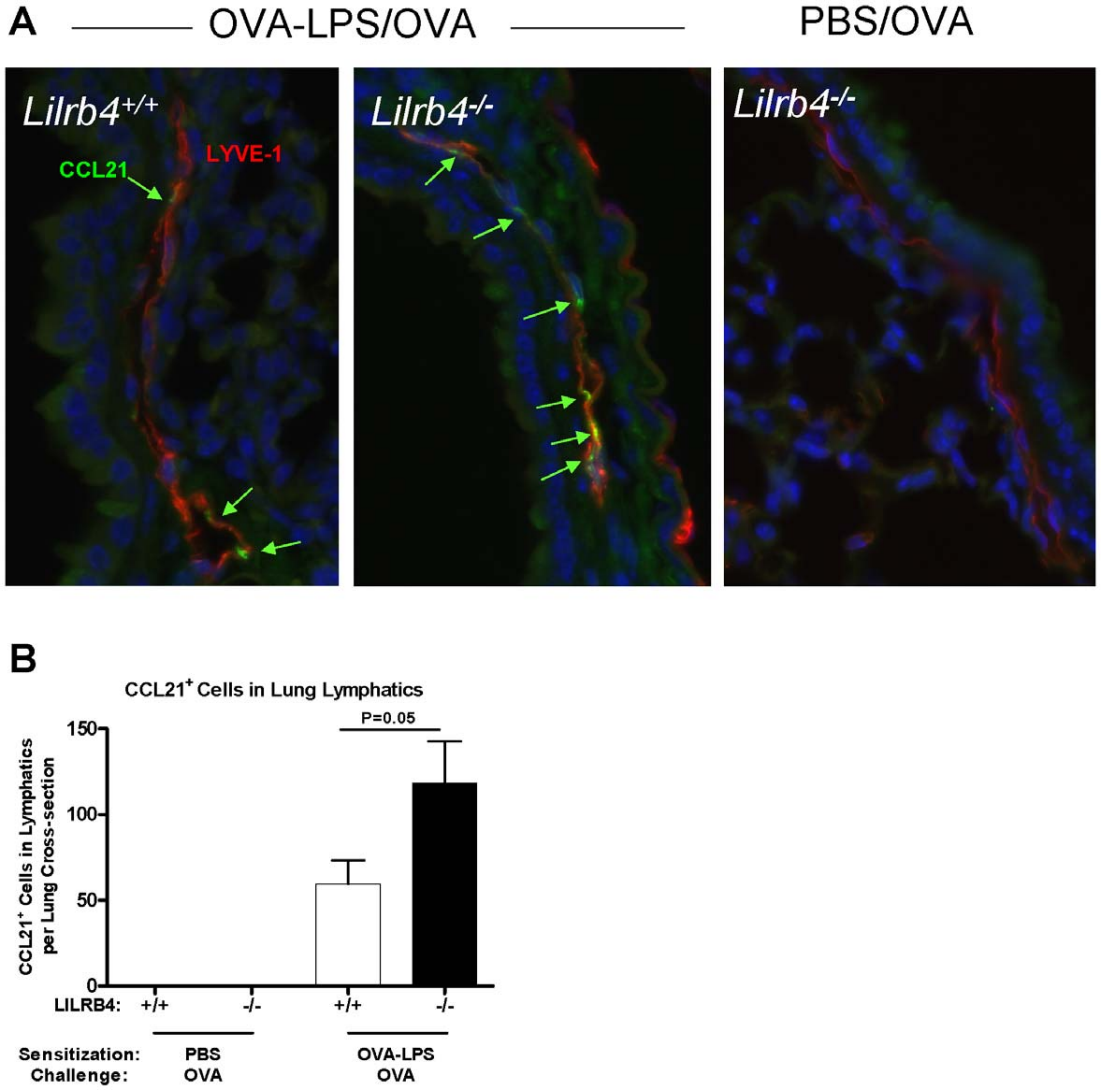
Expression of CCL21 in the lung lymphatics of *Lilrb4^+/+^* and *Lilrb4* ^−/−^ mice. Animals were sensitized with OVA and LPS (*A*; left and middle panels) or sham sensitized with PBS (*A*; right panel) as described in the Fig. 1 legend on days 0, 1, and 2. Mice were challenged with 25 µg of unlabeled OVA intranasally on day 14. After 4 h, mice were euthanized, and paraffin-embedded sections of lungs were prepared. Tissue sections were incubated with rabbit anti-mouse LYVE-1 and goat IgG anti-mouse CCL21. Tissue sections were washed and incubated with Alexa Fluor 488-labeled donkey anti-goat IgG (green), Alexa Fluor 594-labeled chicken anti-rabbit IgG (red), and nuclear dye Hoechst 33342 (blue). Sections were washed, mounted, and examined by fluorescence microscopy. Representative photomicrographs (*A*) and compiled data (*B*) are presented. Data in *B* are expressed as mean ± SEM; *n* = 9–10.

We also previously reported that there are more OVA**^+^**, mature DCs in the lung-draining LNs of OVA/LPS-sensitized *Lilrb4* ^−/−^ mice compared with *Lilrb4^+/+^* mice 18 hours after a single challenge with OVA [16]. To seek a mechanism by which the absence of LILRB4 increases the migration of Ag-bearing DCs from the lungs to the LNs, we considered that CCL21 expressed by cells in the endothelium of lymphatic vessels makes a major contribution to the migration of DCs from tissue sites to local draining LNs [21], [25], [26], [28], [29]. In addition, CCL21 in the lung is located in perivascular lymphatic vessels [22], [23], and Ag-challenged *Lilrb4* ^−/−^ mice have more perivascular lung inflammation than *Lilrb4* ^−/−^ mice [16]. To determine whether CCL21 expression differed in the lungs of *Lilrb4^+/+^* and *Lilrb4* ^−/−^ mice after Ag challenge, mice were sensitized with OVA and LPS or sham sensitized with PBS as described above on days 0, 1, and 2; mice were challenged with 25 µg of unlabeled OVA on day 14. Four hours later, mice were euthanized, lungs were removed, and tissue sections were prepared. As determined by two-color fluorescence immunohistology with anti-LYVE-1 to detect lymphatic vessels [26], [37], [38] and anti-CCL21, immunoreactive CCL21**^+^** cells were not detected in LYVE-1**^+^** lymphatics of mice that were sham sensitized with PBS and then received OVA (representative photomicrographs and quantification are presented in Figs. 2A and 2B, respectively). In contrast, CCL21**^+^** cells were detected in the lymphatic vessels of both *Lilrb4* ***^+/+^*** and *Lilrb4* ^−/−^ mice that had been sensitized with OVA/LPS and challenged with OVA. However, there were more CCL21**^+^** cells in the lymphatics of *Lilrb4* ^−/−^ mice compared with *Lilrb4* ***^+/+^*** mice. In contrast, there were no differences in the number of LYVE-1**^+^** lung lymphatic vessels in *Lilrb4* ***^+/+^*** and *Lilrb4* ^−/−^ mice as assessed immunohistologically, and there were no differences in the levels of lung mRNA encoding LYVE-1, vascular endothelial growth factor C, vascular endothelial growth factor receptor 3, or podoplanin (data not shown), which are molecules that can reflect or influence the production of lymphatic vessels [37], [39], [40]. Hence, the data indicate that the main effect of the absence of LILRB4 is greater expression of CCL21 per lung lymphatic vessel.

### Effects of LILRB4 deficiency on CCR7 expression on lung DCs

**Figure 3:**
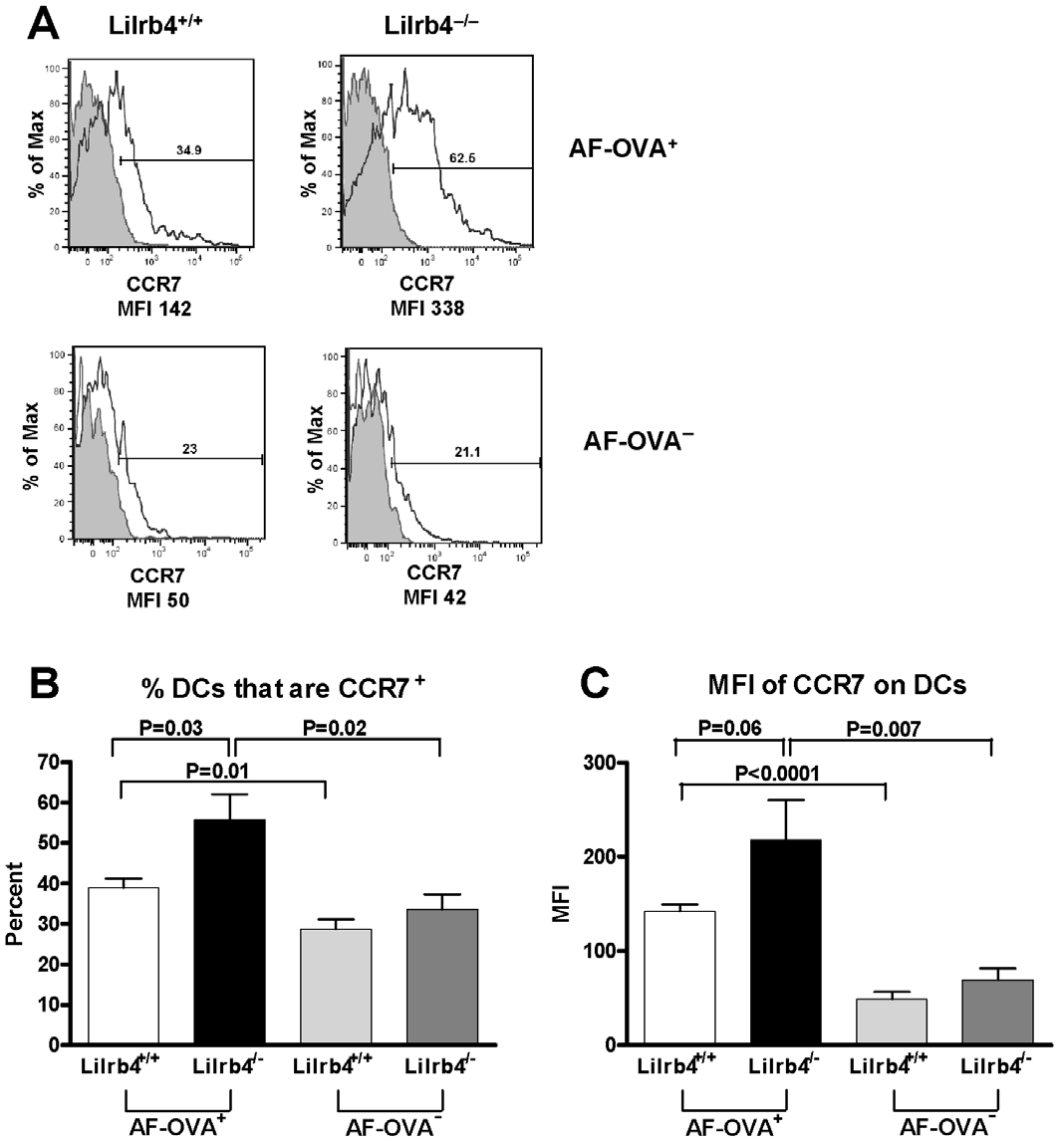
Expression of CCR7 on lung DCs in *Lilrb4^+/+^* and *Lilrb4* ^−/−^ mice. Mice were sensitized with OVA/LPS and challenged with AF-OVA as described in the legend for Fig. 1. After 4 h, lung mononuclear cells were obtained as described in the legend for Fig. 1, and CD11c^+/^autofluorescence**^−^** cells were analyzed by flow cytometry for the percentage (*A*) and MFI (*B*) of CCR7 expression on AF-OVA**^+^** and AF-OVA**^−^** DCs. Fluorescence compensation was set for each color such that there was no cross-talk between detection channels. Data are expressed as mean ± SEM, *n* = 5.

The effects of CCL21 on DC chemotaxis towards, and migration thorough, lymphatics are mediated by CCR7 [22], [24], [27], [29]–[31], including lung DCs carrying inhaled OVA [32]. In addition, upregulation of CCR7 expression on DCs accompanies maturation induced by LPS, TNF-α, and other proinflammatory mediators [33]–[36]. We therefore determined whether expression of CCR7 is greater on Ag-bearing DCs from the lungs of *Lilrb4* ^−/−^ mice at the same time that there is greater lymphatic expression of CCL21, i.e., 4 hours after Ag challenge. *Lilrb4* ***^+/+^*** and *Lilrb4* ^−/−^ mice were sensitized with OVA/LPS as described above and were challenged with AF-OVA on day 14 [16]. Four hours later, lungs cells were mechanically and enzymatically dispersed and mononuclear cells were isolated by density gradient centrifugation. As assessed by flow cytometry, the percentage of AF-OVA**^+^** DCs expressing CCR7 was significantly greater than that of AF-OVA**^−^** DCs in both *Lilrb4* ***^+/+^*** and *Lilrb4* ^−/−^ mice (Fig. 3*A*). However, the percentage AF-OVA**^+^** DCs expressing CCR7 was significantly greater in *Lilrb4* ^−/−^ mice compared with *Lilrb4* ***^+/+^*** mice, whereas there was no difference in the percentage of AF-OVA**^−^** DCs expressing CCR7 in the two strains of mice. Similarly, the expression level of CCR7 as assessed by MFI was significantly greater in AF-OVA**^+^** cells than in AF-OVA**^−^** DCs in both strains of mice (Fig. 3*B*). In addition, there was a trend (P = 0.06) towards greater expression of CCR7 on AF-OVA^+^ DCs but not AF-OVA**^−^** DCs from *Lilrb4* ^−/−^ mice compared with *Lilrb4^+/+^* mice. Hence, ingestion of inhaled Ag by lung DCs resulted in upregulation of the CCL21 ligand CCR7 in both strains, but the increases were greater in *Lilrb4* ^−/−^ mice and were restricted to Ag^+^ DCs. The selectivity of the phenotype for AF-OVA**^+^** cells is in accordance with our finding that OVA**^+^** lung DCs express significantly more LILRB4 than OVA**^−^** DCs in *Lilrb4* ***^+/+^*** mice (Fig. 1).

### Effects of LILRB4 deficiency on Th2 cells in the lung

**Figure 4:**
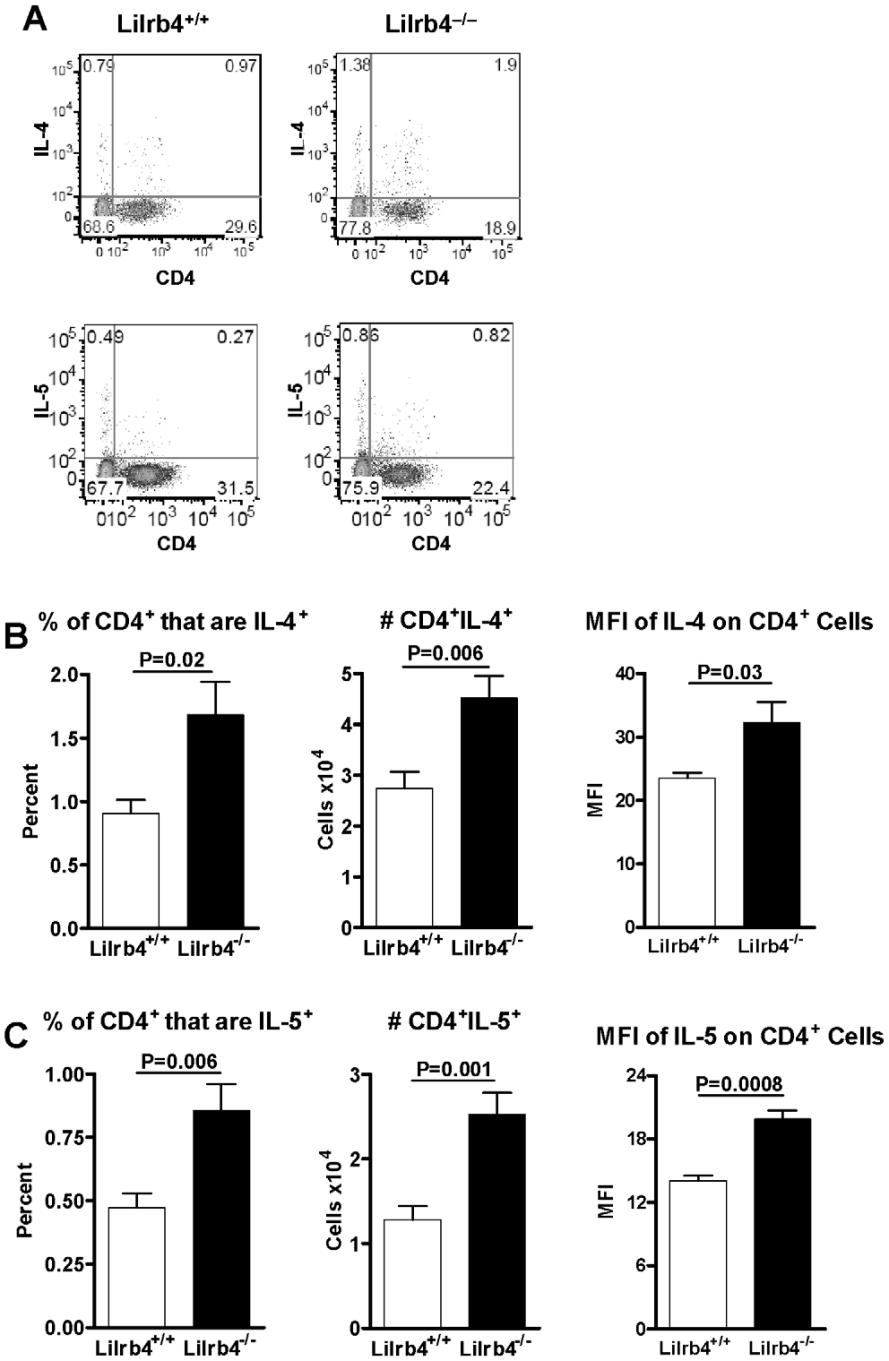
Expression of IL-4 and IL-5 by lung CD4^+^ cells in *Lilrb4^+/+^* and *Lilrb4* ^−/−^ mice. Mice were sensitized and challenged with OVA as described in the legend for Fig. 2. After 18 h, lung mononuclear cells were obtained as described in the legend for Fig. 1, and cells were analyzed by intracellular flow cytometry to determine the percentage, number, and MFI of IL-4 and IL-5 in CD4**^+^** cells. Data from representative experiments show dot plots for IL-4 and IL-5 (A); positivity was defined as cells that had fluorescence intensities greater than that of 99% of the same population of cells when stained with an equal amount of isotype control mAbs. Compiled data for IL-4 (B) and IL-5 (C) are expressed as mean ± SEM, *n* = 8.

We previously found that in addition to the greater number of Ag-bearing DCs in the LNs of *Lilrb4* ^−/−^ mice, there were also more IL-4-producing and IL-5-producing lymphocytes 18 hours after challenge, compared with *Lilrb4* ***^+/+^*** mice ([16] and unpublished data). To determine whether the Th2 phenotype in the LNs was accompanied by greater numbers of IL-4- and IL-5-producing cells in the lungs of *Lilrb4* ^−/−^ mice, we compared by flow cytometry the expression of those cytokines in CD4**^+^** cells in the lungs of sensitized *Lilrb4^+/+^* and *Lilrb4* ^−/−^ mice 18 hours after challenge. Both the percentage (Fig. 4A and B) and number of CD4^+^IL-4^+^ cells were significantly greater in the lungs of *Lilrb4* ^−/−^ mice, as was the MFI of anti-IL-4 staining (Fig. 4B). Although the percentage (Fig. 4A and C) and number of CD4^+^IL-5^+^ cells were approximately half that of IL-4^+^ cells, they were nevertheless significantly greater in *Lilrb4* ^−/−^ mice, as was the MFI of IL-5 staining (Fig. 4B and 4C). These increases in the lungs of challenged *Lilrb4* ^−/−^ mice are consistent with the exacerbated Th2 pulmonary pathology they exhibit [16].

## Discussion

We have established that the absence of LILRB4 leads to changes in lung lymphatic endothelium and DCs that are compatible with the increased migration of Ag-bearing DCs to LNs and attendant increases in Th2 cells in LNs and lungs upon challenge of Ag-sensitized *Lilrb4* ^−/−^ mice. Specifically, our findings of greater expression of CCL21 on lung lymphatics and CCR7 on lung DCs of *Lilrb4^−/−^* mice 4 hours after Ag challenge provides a mechanism by which there are more Ag-bearing DCs and Th2 cells in the LNs of *Lilrb4* ^−/−^ mice 18 hours after challenge. More broadly, the results reported here establish that an ITIM-bearing receptor can exert inhibitory effects by downregulating the expression of both a stromal chemokine and its cognate receptor on immune system cells, leading to attenuated cell migration and resulting pathologic allergic inflammation.

We previously reported that LILRB4 was expressed weakly on a minority of DCs in the lung-draining LNs of *Lilrb4* ^−/−^ mice 18 hours after intranasal instillation of PBS [16]. Inhalation of AF-OVA and LPS resulted in ∼2- and 4-fold increases in the percentage and number of LILRB4**^+^** LN DCs, respectively, compared with mice that received PBS, but notably the increases were entirely accounted for by OVA**^+^** DCs. However, it had not been determined whether the increased expression of LILRB4 occurred before or after DCs arrived in the LNs. We now report that the selective upregulation of LILRB4 on Ag-bearing DCs occurs in the lungs of mice exposed to AF-OVA and LPS intranasally (Fig. 1). These findings are consistent with a lung origin for Ag-bearing LN DCs in response to inhaled OVA and LPS, and demonstrate the retention of upregulated expression of LILRB4 as Ag-bearing DCs migrate from the lungs to the LNs. The finding that upregulation of LILRB4 on DCs occurs proximally in the tissue of origin on the cells destined to attract Ag-specific T cells in the LNs indicates that LILRB4 expression represents an early innate regulatory mechanism that is selectively induced on the DCs that will shape immune and inflammatory responses.

CD11c**^+^**/autofluorescence^+^ cells, characteristic of alveolar macrophages (the population is marked with an asterisk in Fig. 1B), were largely LILRB4^+^ irrespective of whether mice had been treated with PBS or with OVA and LPS (94% and 96%, respectively), in contrast with DCs that exhibited an increase in the percentage of LILRB4^+^ cells from 55% to 84%. Nevertheless, the level of expression of LILRB4 on alveolar macrophages was augmented in response to OVA-LPS compared with PBS (MFI values of 9,303 and 3,924, respectively. In mice that received AF-OVA and LPS, essentially all alveolar macrophages were AF-OVA**^+^** with an LILRB4 MFI of 9,895±244 (*n* = 3), a value similar to that of alveolar macrophages from mice that received unlabeled OVA and LPS. Hence, expression of LILRB4 is upregulated on both Ag-bearing DCs and macrophages in the lungs of mice in response to OVA and LPS. In addition, the finding of comparable MFIs for LILRB4 expression on macrophages in response to unlabeled OVA/LPS and AF-OVA/LPS indicates that the increased MFIs for LILRB4 are not due to the fluorescence of AF-OVA being reported as expression of LILRB4.

We previously reported that LPS increases the expression of LILRB4 on neutrophils *in vivo* [13]. We have also found that LPS increases the MFI of LILRB4 on bone marrow-derived dendritic cells (BMDCs) *in vitro* by approximately 2-fold, a magnitude similar to that for BMDCs cultured in OVA and LPS (data not shown), suggesting that LPS plays a key role in stimulating increased expression of LILRB4 on mouse DCs.

In *Lilrb4* ^−/−^ mice, the major DC phenotype in this model occurs in response to Ag challenge, i.e., greater numbers of Ag-bearing DCs in the lung-draining LNs 18 hours after Ag challenge compared with *Lilrb4^+/+^* mice [16]. However, the mechanism by which LILRB4 regulates the number of Ag-bearing in the LNs was unknown. We found that expression of the key DC migration chemokine CCL21 [21]–[27] is significantly greater in the lung lymphatic vessels [22], [23] of these mice 4 hours after challenge compared with their *Lilrb4^+/+^* counterparts (Fig. 2). Little expression of immunoreactive CCL21 was detected on lymphatic vessels of non-sensitized mice exposed to OVA (Fig. 2). Notably, the immunoreactive CCL21 in sensitized and challenged mice presented in a “patchy” appearance, which has been observed by others [22], [23], [26], [27], and may represent the recently-appreciated localization of CCL21 at basement membrane portals and “flap” structures on lymphatics where the chemokine fosters docking of migrating DCs, their migration into the vessel lumen, and subsequent movement through the vessels [27], [41]. The involvement of CCL21 in multiple steps of DC migration from tissue to lymphatics to LNs emphasizes the key role of this chemokine, and provides an explanation as to how increased expression of CCL21 in *Lilrb4* ^−/−^ mice can contribute to the increased number of Ag-bearing DCs that appear in the LNs.

Expression of CCL21 on lymphatic endothelium is upregulated by mediators associated with inflammation, such as TNF-α, IL-1α [30] lymphotoxin-α1β2 [42], [43], and Oncostatin M [28]. We found no differences in the levels of TNF-α, IL-1α, and Oncostatin M mRNA or protein in whole lung extracts and in isolated lung mononuclear cells from sensitized *Lilrb4* ***^+/+^*** and *Lilrb4* ***^−/−^*** mice 4 hours after challenge, as well as no difference in lymphotoxin-β as determined by immunohistology (data not shown), suggesting that the levels of these mediators in the lung do not account for the enhanced expression of CCL21 in the pulmonary lymphatics of *Lilrb4* ***^−/−^*** mice. It has recently been reported that increased drainage of interstitial fluid through lymphatic vessels increases lymphatic expression of CCL21 [44], [45]. We have previously reported that the absence of LILRB4 leads to increased vascular permeability and tissue edema in two models of allergic responses in the skin [46], [47], raising the possibility that a similar response in the lung might account for the heightened level of lymphatic CCL21 in *Lilrb4^−/−^* mice.

The contribution of upregulated CCL21 in *Lilrb4* ^−/−^ mice with respect to DC migration that we report here is reinforced by the findings that CCR7 expressed on tissue DCs can be an essential component in the mobilization of these cells through lymphatics [22], [24], [27], [29]–[31], particularly as CCR7 is the sole known receptor for CCL21. We found that expression of CCR7 is significantly greater on OVA**^+^** lung DCs compared with OVA**^−^** DCs in response to challenge in both *Lilrb4* ***^+/+^*** and *Lilrb4^−/−^* mice (Fig. 3), demonstrating that OVA**^+^** DCs were primed for directed migration towards, and entry into, CCL21**^+^** lymphatics. Moreover, we found that expression of CCR7 was significantly upregulated in sensitized and challenged *Lilrb4* ^−/−^ mice compared with *Lilrb4* ***^+/+^*** mice (Fig. 3), which together with the increased expression of CCL21, would provide enhanced capacity for migration of Ag-bearing DCs in the absence of LILRB4. Expression of CCR7 on DCs is regulated by cytokines such as IL-33 [48] and transforming growth factor-β1 [49], as well as by prostaglandin (PG) E2 [57;58], but we found no differences in the levels of these cytokines or the PGE2 biosynthetic enzymes cyclooxygenase-1, cyclooxygenase-2, and membrane PGE synthetase 1 in the lungs of sensitized *Lilrb4^+/+^* and *Lilrb4^−/−^* mice after challenge (data not shown). Because expression of CCR7 on DCs is upregulated by LPS [33]–[36], our findings in *Lilrb4^−/−^* mice may therefore represent a direct manifestation of the loss this endogenous negative regulator of LPS responses on DCs.

The ability of a member of the human LILRB family to downregulate expression of a chemokine receptor has been reported for T cells that express LILRB1, the only human LILRB expressed on that cell type. Expression of CXCR3 on human CD4**^+^** T cells is reduced by exposure to soluble HLA-G (sHLA-G) *in vitro* in a manner partially dependent on LILRB1, which is one of the receptors for HLA-G [50]. sHLA-G-mediated downregulation of CXCR3 expression on human NK cells *in vitro* is also regulated in part by LILRB1 [51]. Our findings indicate that mouse LILRB4 not only downregulates the extent of chemokine receptor expression on DCs, but also is a biologically relevant ligand *in vivo*.

Our prior studies indicated that the absence of LILRB4 increases the number of IL-4-producing LN lymphocytes after challenge of sensitized mice [16]. We also reported that the lungs of *Lilrb4* ^−/−^ contained significantly more CD4**^+^** cells, but it was unknown whether those additional cells provided Th2 cytokines. Our new data reveal that in the absence of LILRB4, there are significantly more IL-4-producing and IL-5-producing CD4**^+^** cells in the Ag-challenged lungs of *Lilrb4* ^−/−^ mice (Fig. 4). Because T cells do not express LILRB4 in this model [16], it seems reasonable to conclude that a driving force for the augmentation of cells expressing Th2-cytokines in the LNs and lungs of *Lilrb4* ^−/−^ mice are the Ag-bearing lung DCs that lack the upregulated expression of LILRB4 found in *Lilrb4^+/+^* mice, and their increased migration to LNs under the aegis of increased CCL21 and CCR7 expression. Knock-down of LILRB4 expression in human DCs leads to greater production of the T cell chemoattractants CXCL10 (IP-10) and CXCL11 (I-TAC) in response to LPS-induced maturation, and supernatants from these DCs promote greater *in vitro* chemotaxis of activated T cells than supernatants from DCs that express normal levels of LILRB4 [52], suggesting another mechanism by which LILRB4 on DCs may regulate the translation of an innate response to an adaptive response by inhibiting cell chemotaxis. Overall, our findings establish that in the context of a model of pulmonary allergic inflammation, LILRB4 downregulates DC-T cell interactions by inhibiting the chemotaxis/migration of DCs from lung to secondary lymphoid tissue and mitigating the pathobiologic linkage of innate and adaptive immune inflammation.
